# Spectrophotometric Estimation of Bicalutamide in Tablets

**DOI:** 10.4103/0250-474X.49131

**Published:** 2008

**Authors:** P. P. Sancheti, V. M. Vyas, Manali Shah, Poonam Karekar, Y. V. Pore

**Affiliations:** Government College of Pharmacy, Vidyanagar, Karad-415 124, India

**Keywords:** Bicalutamide, UV spectroscopy, tablet dosage forms

## Abstract

A simple, sensitive, rapid, accurate and precise spectrophotometric method has been developed for the estimation of bicalutamide in bulk and pharmaceutical dosage forms. Bicalutamide shows maximum absorbance at 272 nm with molar absorptivity of 2.3399×10^4^ l/mol/cm. Beer's law was obeyed in the concentration range of 1.5-18 μg/ml. The limit of detection and limit of quantification were found to be 0.1 and 0.4 μg/ml, respectively. Results of analysis were validated statistically and by recovery studies.

Bicalutamide, chemically, (2RS)-4'-cyano-3-(4-fluorophenylsulphonyl)-2-hydroxy-2-methyl-3'-(trifluoromethyl)-propionanilide is an orally active, nonsteroidal antiandrogen^1^. It is mainly used in the treatment of prostate cancer^2^. It competitively blocks the growth-stimulating effects of androgens on prostate tumors^3^. The antiandrogenic activity resides almost exclusively in (*R*)-bicalutamide with little activity in (*S*)-bicalutamide^4^–^6^. It is highly lipophilic drug (log P 2.92) having very low aqueous solubility (5 mg/l)^3^. Literature survey revealed that the stability indicating liquid chromatographic method is available for the quantitative estimation of bicalutamide in bulk drugs^7^. The spectral characteristics of bicalutamide drug in different solvents and aqueous β-cyclodextrin has been also reported^8^. However, no UV spectrophotometric method is available for the quantitative determination of bicalutamide in its pharmaceutical dosage forms.

This work was aimed to develop simple, rapid, accurate and specific UV spectrophotometric method for the estimation of bicalutamide in pharmaceutical dosage forms. The method was further validated for the parameters like precision, accuracy, sensitivity, and linearity. The limit of detection (LOD) and limit of quantification (LOQ) were also determined. The results of analysis were validated statistically and by recovery studies. This method of estimation of bicalutamide was found to be simple, precise and accurate.

Bicalutamide was obtained as a gift sample from Lupin Ltd., Mumbai, India. Bicalutamide tablets were procured from local pharmacy. All the reagents were of analytical grade. Double distilled water was used throughout the experiment. A GBC UV/Vis 911 A spectrophotometer with 1 cm matched quartz cells were used for the estimation.

An accurately weighed 5 mg of bicalutamide was dissolved in 5 ml of dimethylformamide (DMF) in a 50 ml volumetric flask and the volume was adjusted up to the mark with 1% sodium lauryl sulphate (SLS) prepared in distilled water to obtain a stock solution of 100 μg/ml. Aliquots of 0.15 to 1.8 ml portions of standard solution were transferred to a series of 10 ml volumetric flasks and volume in each flask were adjusted to 10 ml with 1% SLS to obtain concentration of range of 1.5-18 μg/ml. One of the solutions was scanned in UV range using DMF: 1% SLS (1:9) as a blank and λ_max_ was found to be 272 nm. The absorbance of solutions was measured at 272 nm against reagent blank and calibration curve of bicalutamide was constructed. The optical characteristics are presented in Table 1.

**TABLE 1:** OPTICAL CHARACTERISTICS AND REGRESSION EQUATION FOR THE STANDARD BICALUTAMIDE

Parameter	Value
λ_max_ (nm)	272
Beer's range (μg/ml)	1.5-18
Molar absorptivity (l/mol/cm)	2.3399×10^4^
Sandell's sensitivity (μg/cm^2^/0.001AU)	0.018392
Correlation coefficient (r^2^)	0.9988
Regression equation	y = 0.054371× + 0.040606
Intercept (a)	0.040606
Slope (b)	0.054371
Limit of detection (LOD μg/ml)	0.1
Limit of quantification (LOQ μg/ml)	0.4

Twenty tablets of bicalutamide were weighed and powered in glass mortar. Amount equivalent to 5 mg was transferred to 50 ml volumetric flask, dissolved in 5 ml of DMF and made up the volume with 1% SLS to obtain a concentration of 100 μg/ml. The solution was filtered through Whatman filter paper No. 41 and filtrate was diluted to obtain concentration in between linearity range. The absorbance of sample solution was measured and amount of bicalutamide was determined by referring to the calibration curve. Recovery studies were carried out by adding a known quantity of pure drug to the preanalyzed formulation and the proposed method was followed. From the amount of drug found, percentage recovery was calculated. The results obtained are given in Table 2.

**TABLE 2:** RESULTS OF ANALYSIS AND RECOVERY STUDIES

Formulations	Label Claim mg	% Estimated	SD	COV (%)	SE	% Recovery
Calutide	50	98.82	0.27	0.28	0.16	98.63

SD is standard deviation, SE is standard error and COV is coefficient of variation

The proposed method of determination of bicalutamide showed molar absorptivity of 2.3399×10^4^ l/mol/cm and Sandell's sensitivity 0.018392 μg/sq.cm/0.001-absorbance units. Linear regression of absorbance on concentration gave equation y=0.054371x+0.040606 with a correlation coefficient of 0.9988. Relative standard deviation of 0.002346 was observed for analysis of 3 replicate samples, indicating precision and reproducibility. Bicalutamide exhibits its maximum absorption at 272 nm and obeyed Beer's law in the range of 1.5-18 μg/ml. Limit of detection (LOD) and limit of quantification (LOQ) were calculated by Eqs. 1, LOD= 3.3 δ/s and 2, LOQ= 10 δ/s, respectively, where δ is the standard deviation of blank and s is slope of calibration^9^.

The LOD and LOQ were found to be 0.1 μg/ml and 0.4 μg/ml, respectively. The results of analysis and recovery studies are presented in the Table 2. The percentage recovery value 98.63% indicates that there is no interference from the excipients present in formulation. The developed method was found to be sensitive, accurate, precise and reproducible and can be used for the routine quality control analysis of bicalutamide in bulk drugs and formulations.
